# A hybrid method for the imputation of genomic data in livestock populations

**DOI:** 10.1186/s12711-017-0300-y

**Published:** 2017-03-03

**Authors:** Roberto Antolín, Carl Nettelblad, Gregor Gorjanc, Daniel Money, John M. Hickey

**Affiliations:** 1 The Roslin Institute and Royal (Dick) School of Veterinary Studies, The University of Edinburgh, Easter Bush Research Centre, Midlothian, EH25 9RG Scotland, UK; 2 Division of Scientific Computing, Department of Information Technology, Science for Life Laboratory, Uppsala University, Lägerhyddsvägen 2, Box 337, 751 05 Uppsala, Sweden

## Abstract

**Background:**

This paper describes a combined heuristic and hidden Markov model (HMM) method to accurately impute missing genotypes in livestock datasets. Genomic selection in breeding programs requires high-density genotyping of many individuals, making algorithms that economically generate this information crucial. There are two common classes of imputation methods, heuristic methods and probabilistic methods, the latter being largely based on hidden Markov models. Heuristic methods are robust, but fail to impute markers in regions where the thresholds of heuristic rules are not met, or the pedigree is inconsistent. Hidden Markov models are probabilistic methods which typically do not require specific family structures or pedigree information, making them very flexible, but they are computationally expensive and, in some cases, less accurate.

**Results:**

We implemented a new hybrid imputation method that combined heuristic and HMM methods, AlphaImpute and MaCH, and compared the computation time and imputation accuracy of the three methods. AlphaImpute was the fastest, followed by the hybrid method and then the HMM. The computation time of the hybrid method and the HMM increased linearly with the number of iterations used in the hidden Markov model, however, the computation time of the hybrid method increased almost linearly and that of the HMM quadratically with the number of template haplotypes. The hybrid method was the most accurate imputation method for low-density panels when pedigree information was missing, especially if minor allele frequency was also low. The accuracy of the hybrid method and the HMM increased with the number of template haplotypes. The imputation accuracy of all three methods increased with the marker density of the low-density panels. Excluding the pedigree information reduced imputation accuracy for the hybrid method and AlphaImpute. Finally, the imputation accuracy of the three methods decreased with decreasing minor allele frequency.

**Conclusions:**

The hybrid heuristic and probabilistic imputation method is able to impute all markers for all individuals in a population, as the HMM. The hybrid method is usually more accurate and never significantly less accurate than a purely heuristic method or a purely probabilistic method and is faster than a standard probabilistic method.

**Electronic supplementary material:**

The online version of this article (doi:10.1186/s12711-017-0300-y) contains supplementary material, which is available to authorized users.

## Background

This paper describes a combined heuristic and hidden Markov model (HMM) method to accurately impute missing genotypes in livestock datasets. Methods for imputing genotypes are essential for modern livestock breeding because they help to facilitate genomic selection, which has become the dominant method for genetic evaluation of livestock. Imputation can cost-effectively generate the high-density genotypes of many individuals required for genomic selection [1, 2]. Typically, the genotyping strategies used in livestock breeding involve genotyping a small number of individuals with expensive high-density marker panels and large numbers with cheaper low-density panels, then using imputation to infer the untyped high-density markers in the individuals genotyped at low-density. Imputation methods work by identifying haplotypes shared between individuals. The methods used generally fall into two broad categories: (1) heuristic methods that are designed to identify and propagate linkage information about long haplotypes (e.g., >10 cM), which is typically shared between closely related individuals; and (2) probabilistic methods that are designed to identify and propagate linkage disequilibrium information about short haplotypes (e.g., <1 cM), which is typically shared between distantly related individuals.

Heuristic methods use the basic principles of inheritance and are fast and accurate in many of the circumstances that are common to livestock applications [3]. Heuristic methods make explicit use of pedigree information and make inferences from information on closely related individuals from large families and the large portions of the genome shared between pairs of related individuals. However, heuristic methods do not impute alleles if such data is lacking or unreliable. The AlphaImpute program [3] is an example that combines several heuristic methods, such as basic rules of Mendelian inheritance, long-range phasing, and haplotype library imputation algorithm [4]. Other examples that are based on heuristic methods include Findhap [5] and FImpute [6].

Probabilistic methods mainly use HMM approaches to model genotype variation along chromosomes and the sharing of genomic segments between nominally unrelated individuals. HMM-based imputation methods are computationally more demanding, slower, and inherently less accurate than heuristic methods when they do not take pedigree information into account. HMM methods commonly used in livestock applications were primarily developed for application in human populations where pedigree information is typically lacking and pedigree structures, such as small family sizes, are not well-suited for exploiting heuristic algorithms.

HMM methods are used to describe the variation of an observable variable in a sequence, as a function of an underlying sequence of hidden variables that each have a set of $K$ distinct states [7]. When HMM methods are applied to genotype imputation, the observable variable is a marker genotype, the sequence is a set of $M$ markers along the chromosome, and the hidden variable represents the possible haplotypes that underlie the genotype. Given the number of markers, $M$, and the number of hidden states, $K$, the computational time of hidden Markov models scale as $O\left( {M \times K^{2} } \right),$ which limits the effectiveness of genomic applications with large numbers of markers and many possible haplotypes.

Distinct HMM algorithms with different representation of hidden states and computational considerations have been developed to alleviate the computational burden when analysing dense genomic data, such as, PHASE [8]; fastPHASE [9]; Beagle [10]; SHAPE-IT [11]; Impute2 [12]; MaCH [13]; MERLIN [14]; cnF2freq [15]. PHASE uses a Markov chain Monte Carlo algorithm to estimate the actual pair of hidden gametes of each individual as a mosaic of haplotypes given the observed genotypes and the underlying recombination rates [8]. PHASE is very accurate but computationally intractable for large datasets. fastPHASE, Beagle, SHAPE-IT, Impute2, and MaCH are computationally tractable HMM methods for phasing and imputation. fastPHASE uses an expectation–maximisation approach and infers the most likely hidden states by clustering similar haplotypes [9]. Albeit faster, fastPHASE is still computationally expensive and its expectation–maximisation algorithm can get stuck in a local maximum. Beagle relies on a similar concept as fastPHASE, but clusters haplotypes locally [10]. SHAPE-IT follows the HMM of PHASE but collapses all the haplotypes into a graph structure and uses this structure to divide the haplotypes into disjoint segments of *J* distinct haplotypes used as hidden states [11]. SHAPE-IT samples pairs of haplotypes with a Markov chain Monte Carlo algorithm that is linear in the number of the distinct haplotypes, *J*, to reduce computational intensity even further. SHAPE-IT is also able to integrate pedigree information for disjoint sets of duos and trios directly in the model, as well as adding a proof-reading step based on the separate local duo-HMM model [16]. Impute2 approximates PHASE but, instead of conditioning on all haplotypes of all individuals, it restricts the number of haplotypes to the effective population size to decrease computational intensity [12]. Impute2 selects haplotypes that are similar to the haplotypes of the individual being imputed as the hidden states, which can lead to local minima [17].

MaCH is close to PHASE in the use of a mosaic of haplotypes to explain the observed genotypes. However, MaCH uses a model parameterised by recombination and mutation rates to iteratively improve the phasing of each individual in a Markov chain Monte Carlo framework [13]. In this regard, MaCH is similar to Impute2, but the method selects a user specified number of template haplotypes at random instead of selecting those that are expected to be similar to haplotypes carried by the individual being imputed as in the case of Impute2. To reduce computational intensity, MaCH limits the template haplotypes, to a number specified by the user. MERLIN models the state space as the combination of haplotypes of all individuals that are included in the same pedigree [14]. This is successful for small nuclear families, but it is not feasible for livestock applications where the number of hidden states increases exponentially with the number of individuals. However, in the cases it can handle, the optimum solution achieved is the globally preferable solution based on the modelling assumptions, while most other HMM approaches only provide approximations. cnF2freq models the state of multiple individuals at once, but maintains several separate local pedigrees of a single individual and its immediate ancestors, making the problem tractable, but again introducing the risk of getting stuck in a local optimum [15].

Heuristic and HMM imputation methods have different strengths and weaknesses. Combining the two approaches in a single algorithm may improve performance. Errors and missing genotypes generated by heuristics can be resolved by exploiting the probabilistic nature of Markov models. For example, if one parent of an individual is known, genotyped, and can be fully phased using heuristics, while the other parent is unknown, not genotyped, or cannot be phased using heuristics, a heuristic method can be used to impute the alleles on the gamete inherited from the first parent, while a HMM can be used to impute the alleles on the gamete inherited from the other parent.

Heuristic methods can also be used to increase the computational efficiency of HMM methods [18]. The phased information from the heuristic approach, which can be obtained with a very limited computational burden, can be used to supply the HMM with an accurate, relevant and relatively small set of reference haplotypes. This set of reference haplotypes can reduce the computational demand of the HMM permitting it to handle large populations better. Similar ideas are used in minimac, which achieves very fast computation by using pre-phased data [19].

In this study, we present a combined method for genotype imputation that takes advantage of the accuracy and speed provided by heuristic methods and the robustness of HMM methods. In particular, the method improves the heuristic method of AlphaImpute [3] with the HMM implemented in MaCH [13], and has been released as the version v1.5 of AlphaImpute. Performance of the combined method using real and simulated data is compared with results obtained separately from heuristic and HMM methods.

## Methods

The imputation method presented in this study is a combined heuristic and HMM method to impute genotypes of all individuals in a population for all markers. The method incorporates the heuristic imputation approach of AlphaImpute and the HMM method of MaCH. AlphaImpute implements the heuristic method explained in Hickey et al. [3] and MaCH implements the HMM explained in Li et al. [13]. All three methods are briefly described in the following sections.

### Heuristic method of AlphaImpute

AlphaImpute combines: (1) basic rules of Mendelian inheritance; (2) segregation analysis; (3) long-range phasing; and (4) haplotype library imputation in order to phase and impute genotype data of all individuals in a population [3]. The program iterates across these four sets of actions multiple times to accumulate information and determine the haplotype that each individual carries at each position along the genome.

The basic rules of Mendelian inheritance and the segregation analysis are used in conjunction with all pedigree and genotype information to derive phase for as many alleles as possible under the assumption that each locus is inherited independently of its neighbours at this step.

Long-range phasing and haplotype library imputation, which are both implemented in the AlphaPhase software and described in full detail in Hickey et al. [4], are used to derive the haplotypes that are carried by the individuals that are genotyped at high-density. Both long-range phasing and haplotype library imputation work by dividing the genome into genome regions, referred to as cores, and resolving the haplotypes within the cores for the individuals concerned. Cores of different lengths are used in several runs to phase each locus as part of overlapping cores and to facilitate the identification of phasing errors. These phasing steps generate a library of haplotypes for each core that are used later.

Missing alleles are then imputed by matching haplotypes that are obtained during the long-range phasing to alleles that are imputed and phased by the basic rules of Mendelian inheritance and by the segregation analysis. All haplotypes stored in the haplotype libraries are considered candidates of the true haplotype of the proband (i.e., the individual being phased) for each core of each phasing round. Alleles that are imputed and phased by the basic rules of Mendelian inheritance and by the segregation analysis are compared to corresponding alleles in each of the haplotypes in the library and haplotypes that are consistent with the alleles of a proband are retained as candidate haplotypes. This is repeated for each core. For a given marker position, individual alleles are imputed such that all remaining haplotypes across all of the cores that span this marker are in agreement. To impute from parental haplotypes this process is also repeated with a restriction that the haplotypes retained in the haplotype library comprise only those haplotypes that are carried by the parents. Libraries are updated with any new haplotype found. The matching process is iterated a defined number of times and at the end of each iteration each chromosome of each individual is traversed in each direction to detect recombination locations and to model the imputation of alleles in the regions of uncertainty that are adjacent to these recombinations as a weighted average of the two gametes carried by the relevant parent [20]. At the end of the final iteration, the segregation analysis is repeated and used to fill in alleles that remain unimputed.

### Hidden Markov model algorithm of MaCH

In its diploid form, MaCH implements a HMM that characterises the unphased genotypes, $\varvec{G},$ as a mosaic of pairs of haplotypes taken from a set of template haplotypes, $\varvec{H}$ [13]. The mosaic of pairs of haplotypes represents the hidden sequence of states. At a locus, $i = 1, \ldots ,M,$ a hidden state is represented as $S_{i} = \left( {h_{i} ,k_{i} } \right),$ where *h*_i_ corresponds to the haplotype on the first gamete, and $k_{i}$ corresponds to the haplotype on the second gamete. The total number of possible states is $\left| \varvec{H} \right|^{2} ,$ corresponding to all the combinations of pairs of haplotypes, $\varvec{S} = \{ \left( {h, k} \right) \left| {h, k \in H\} } \right.,$ where $h$ and $k$ are two haplotypes of $\varvec{H}.$ The objective is to deduce the hidden sequence of states that best fits the data, i.e., to estimate the best pair of haplotypes that explain the unphased observed genotypes. This information in turn enables imputation at the unobserved loci.

In the HHM implemented in MaCH:

1. the prior probability is defined by assuming that every state is equally likely at the first marker,
2. the probability of transition from one state to another is given as a function of $m - 1$ crossover parameters, $\theta_{i} ,$ which reflect the recombination rates, and
3. the probability of observing a genotype at each locus given a particular state is defined as a function of the *m* error parameters, $\varepsilon_{i} ,$ which indicate the effect of genotype errors and mutations.

MaCH uses a Monte-Carlo procedure to estimate the best pair of haplotypes for each individual [13]. Initially, the program samples a pair of haplotypes that is compatible with the observed genotype data. Alleles in the initial pair of haplotypes are phased randomly for heterozygous markers and sampled according to population allele frequencies for markers with missing data. Each individual is then updated with two new haplotypes that are sampled from the template haplotypes. This step involves calculating likelihoods of the hidden states, which is solved using the computationally efficient Baum’s forward–backward algorithm. To limit computational complexity, the set of template haplotypes is represented by a random subset of all the current haplotype estimates.

The updating process is repeated an arbitrary number of iterations over the whole population. Each iteration involves an update of the model parameters (mutation and recombination rates). The crossovers and the errors are set to 0.01 at first, and are re-estimated at each iteration. For this purpose, the algorithm stores the number and position of recombinations, and the number of times the inferred genotype agrees with the observed one. The new estimates of the parameters improve the model and, thus, the haplotype sampling. The final pair of haplotypes for each individual is the consensus pair that minimises the total proportion of switches in haplotypes (i.e., switch error) when compared to the haplotypes sampled at each round.

MaCH also implements a haploid version of the model described above to improve the model when phase data is available. For phased individuals, the Baum’s forward–backward algorithm is used twice to independently sample the two haplotypes from the template haplotypes.

The computational complexity of analysing a single individual in MaCH scales as $O\left( {M \times \left| \varvec{H} \right|^{2} } \right),$ where *M* is the number of markers and $\left| \varvec{H} \right|$ is the number of template haplotypes. The complexity of the forward–backward algorithm is $O\left( {M \times \left| \varvec{S} \right|^{2} } \right),$ where $\left| \varvec{S} \right|$ is the number of hidden states. Therefore, the diploid HMM of the MaCH algorithm scales as $O\left( {M \times \left| \varvec{S} \right|^{2} } \right) = O\left( {M \times |\varvec{H}|^{4} } \right),$ thus the number of template haplotypes is usually limited to reduce computational load. However, it is possible to further reduce the computational requirements of the algorithm to $O\left( {M \times \left| \varvec{H} \right|^{2} } \right)$ by taking advantage of regular patterns within the transition probability matrix.

### Motivation for an algorithm that combines AlphaImpute and a HMM

AlphaImpute is computationally feasible for large datasets and is highly accurate for most of the genome of most individuals in typical livestock populations, but it has some weaknesses. For example, an individual that is to have its high-density genotypes imputed is genotyped at low-density, its sire and maternal grandsire are both genotyped at high-density and there is sufficient high-density information on their relatives to enable their genotype data to be completely and accurately phased. However, no genotype information is available on the dam and the maternal granddam. Thus, this data would enable all of the gametes the individual inherited from its sire to be accurately imputed, with the exception of the few small regions that are adjacent to recombination events. The imputation of the alleles on the gamete inherited from the individual’s dam is more complex. While the portion of this gamete that derives from the maternal grandsire would be imputed with a relatively high level of accuracy, the lack of information available to impute the portion of this gamete that derives from the maternal granddam makes its imputation more difficult. These situations are common in livestock populations, where genotyping strategies often involve only genotyping male ancestors at high-density.

Another common situation is that the genotyping strategy may involve genotyping all parents of selection candidates at high-density and the selection candidates themselves at low-density. The quality control checks of the genotype data may show that one of the parents of a selection candidate is incorrectly identified in the pedigree records and must be set to missing. Thus, AlphaImpute would be able to accurately impute the gamete that the selection candidate inherited from one of its parents, but would have limited ability to perform imputation of the gamete inherited from the parent that has been set to missing. Also an individual that is part of the population being imputed by AlphaImpute may have unknown pedigree information for both its sire and its dam, due to any number of reasons, while most of the rest of the population has complete or partially complete pedigree information. In all three of these examples, a HMM could impute the segments that AlphaImpute is not able to impute well.

HMM methods are computationally intensive and typically less accurate than AlphaImpute in circumstances in which the pedigree information and population structure allow AlphaImpute to perform well. Much of the computational requirements of a HMM, and imputation errors that result from it, derive from establishing the template haplotypes and estimating the recombination rates between markers. AlphaImpute can accurately resolve haplotypes with computational efficiency.

Based on the different strengths, the two methods could be used to augment each other in several ways. For instance, accurate haplotypes that are resolved with heuristic methods could be fed as template haplotypes to a HMM and thus be used to increase its imputation accuracy and computational efficiency. Template haplotypes could be chosen for a particular genome segment with or without regard to any available pedigree information. A haploid version of the HMM could be used for individuals for which AlphaImpute could perform imputation for one gamete but not the other or not for part of one of the gametes while a diploid version of the same model could be used for individuals for which AlphaImpute could not perform imputation for either gamete or for the same region of both gametes. In addition, in some situations the heuristic rules of AlphaImpute may be able to partially impute some of the alleles on a gamete or segment of a gamete. This would effectively increase marker density and could increase the accuracy of the subsequent imputation by the HMM.

### Hybrid algorithm

The method we propose (hybrid method) combines the different components of AlphaImpute with the HMM that underlies MaCH into a single framework. The hybrid method begins by applying the imputation method of AlphaImpute: basic rules of Mendelian inheritance, segregation analysis, long-range phasing, and haplotype library imputation. This gives accurate imputation for all individuals for which enough information is available. It also gives a large haplotype library that includes some whole-chromosome phased gametes. The HMM is then applied to impute the remaining missing alleles of any individual that are not imputed by AlphaImpute. The haplotype library is used to sample the template haplotypes used in the HMM. The haplotype update is run through several iterations so the parameters of the model converge. The solutions of the first iterations are disregarded as burn-in. Allele dosages and the final imputation are calculated using the remaining iterations. Finally, haplotypes are constructed from the most frequent alleles for each locus, and allele probabilities are assessed as the average across runs.

Within the hybrid method we devised three training modes and two imputation modes for the HMM in order to suit particular situations. When applying the method to a dataset, a user can use any combination of these modes:

- *Training mode 1* Use the high-density genotypes in the diploid HMM to estimate the model parameters;
- *Training mode 2* Sample a set of template haplotypes from a haplotype library and use them in the haploid HMM to estimate the model parameters;
- *Training mode 3* Use both of the data types and versions of the HMM from training modes 1 and 2 jointly while ensuring that genomic data for any individual enters the training set only once, with the haplotype information taking precedence;
- *Imputation mode 1* Use the haploid HMM to impute segments of a gamete that have not been imputed by AlphaImpute;
- *Imputation mode 2* Use the diploid HMM to impute segments of both gametes that have not been imputed by AlphaImpute.

To determine which training mode should be used for a given dataset, we defined some heuristic rules. When the user chooses not to use the heuristic method of AlphaImpute, Training mode 1 is used. When the number of phased gametes from AlphaImpute is above a user specified threshold Training mode 2 is used by default. When the number of phased gametes from AlphaImpute is below the user specified threshold Training mode 3 is used by default. Training mode 3 could also be used in datasets with peculiar properties, such as in some F_1_ crossbred datasets in which AlphaImpute has sufficient information to resolve the haplotypes in one of the parental breeds but not in the other.

To determine which imputation mode should be used for a given segment of a gamete or pairs of individuals’ gametes, the proportions of alleles imputed for the individual by AlphaImpute are examined. When the number of imputed alleles of a gamete from AlphaImpute is above a user-specific threshold Imputation mode 1 is used. When the number of imputed alleles of both individuals’ gametes from AlphaImpute is above the user-specific threshold Imputation mode 2 is used.

The hybrid algorithm is designed to work for biallelic markers. Alleles are coded as 0, 1 or 9, where 9 is a missing allele; allele probabilities range from 0 to 1. Genotypes are determined as the sum of the allele codes, and allele dosages are assessed as the sum of the allele codes weighted by the allele probabilities. Therefore, genotypes are coded as 0, 1, 2 or 9, where 0 is the reference homozygote, 1 is the heterozygote, 2 is the alternative homozygote, and 9 is a missing genotype. Allele dosages range continuously from 0 to 2.

### Datasets

Performance of the hybrid method was tested using both a real pig dataset [courtesy of The Pig Improvement Company (PIC)] and simulated data.

#### Real data

In the real pig dataset, genotype information was spread sparsely across multiple generations. The pedigree consisted of 6473 animals, including 3213 genotyped at high-density with the Illumina PorcineSNP60 BeadChip. Some animals had multiple generations of high-density genotyped ancestors available, while others had no, or very few, ancestors genotyped at high-density. This population came from a single PIC breeding line and therefore, animals were moderately to highly related to each other as is typical in most livestock breeding programs.

The genotyped animals were divided into training and testing sets. The testing set comprised the 509 most recently born animals genotyped at high-density. We used a single chromosome with 3129 quality-controlled high-density markers. To explore the effect of different genotyping strategies, animals in the testing set had different number of low-density markers selected from high-density, which were used to impute the remaining markers. We used 15, 30, 300, 600, or 2000 low-density markers on this chromosome, which were selected at random. These numbers are roughly equivalent to 300, 600, 6000, 12,000 and 40,000 markers per 20 chromosome genome. The training set comprised the remaining 2704 animals genotyped at high-density for all markers.

To explore the effect of having genotype data for the ancestors, animals in the testing set were grouped into six categories. These categories represented patterns of relationship between the animals in the population and their most recent high-density ancestors. The categories were: both parents genotyped (Both); sire and maternal grandsire genotyped (SireMGS); dam and paternal grandsire genotyped (DamPGS); sire genotyped (Sire); dam genotyped (Dam); and other relatives genotyped (Other).

#### Simulated data

Two sets of data were also simulated. For the first set, the pedigree of the real pig dataset was used and genotype data were simulated for different high and low-density panels. For the second set, a five-generation pedigree was simulated by mating 25 sires with 500 dams to produce 1000 progeny per generation. Genotype data were simulated for high and low-density panels. To explore the effect of imputation in the absence of pedigree data, another pedigree was created by randomly removing the sire and dam links for 500 individuals in the last generation of the simulated pedigree. These individuals without pedigree information were treated as unrelated individuals and the remaining pedigree information was used for imputing other animals and for resolving haplotypes of the training set. The parent’s genotypes were retained in the training set.

Data for five replicates encompassing different genotyping strategies were simulated. The simulation of the genotype data required the following three steps:

1. Generate whole-genome sequence data,
2. Generate the marker genotypes, and
3. Mask the genotype information for markers that are not in the low-density panels.

Sequence data was generated using the Markovian Coalescent Simulator (MaCS) [21] and AlphaDrop [22] for 1000 base haplotypes for each of 30 chromosomes. Chromosomes were each 100 cM long, comprised 10^8^ base pairs and were simulated using a per site mutation rate of 2.5 × 10^−8^, a per site recombination rate of 1.0 × 10^−8^ and an effective population size that varied over time in accordance with estimates for the Holstein cattle population [23]. The population size was set to 100 in the final generation of the coalescent simulation, to 1256 at 1000 years ago, to 4350 at 10,000 years ago, and to 43,500 at 100,000 years ago, with linear changes in between these time-points. The resulting sequence had approximately 1.7 million segregating sites in total.

Chromosomes of individuals in the first generation were sampled from the 1000 simulated base haplotypes and those in the following generations were sampled from the chromosomes of their parents involving recombination. Crossovers occurred with 1% probability per cM and were uniformly distributed along the chromosomes.

For each chromosome, high-density panels were created by randomly selecting 2000 (H2k) and 10,000 (H10k) of the segregating sites. These numbers are roughly equivalent to 60,000 and 300,000 markers per 30-chromosome genome.

To explore the effect of genotyping strategies, low-density panels were simulated by masking the genotype information of some individuals in the pedigree. For the simulated dataset using the real pig pedigree, we masked the genotypes of the same 509 animals included in the testing set of real pig dataset. For the dataset with the simulated pedigree, we masked the genotypes of the 1000 individuals in the last generation. The genotype information was masked by selecting 15 (L15), 30 (L30), 300 (L300), 600 (L600), and 2000 (L2k) markers at random and removing the genotype information of the remaining markers from the H2k and H10k high-density panels. Low-density panels had densities equivalent to 500, 1000, 9000, 18,000 and 60,000 markers per 30 chromosome genome. A summary of the high and low-density panels is in Table 1.

**Table 1:** Description of the simulated marker panels

Panel code	Panel design	Number of markers per chromosome	Number of markers across the genome
H10k	High-density	10,000	300,000
H2k	High-density	2000	60,000
L2k	80%	2000	60,000
L600^a^	94/70%	600	18,000
L300^a^	97/85%	300	9000
L30^a^	99.7/98.3%	30	900
L15^a^	99.8/99.2%	15	450

Low-density panels were simulated by randomly masking the genotype information of the H10k and H2k high-density panels. 15, 30, 300, 600, and 2000 markers per chromosome were selected at random from both H10k and H2k high-density panels, to simulate densities equivalent to 450, 900, 9000, 18,000 and 60,000 markers per 30 chromosome genome

^a^Values correspond to percentage of markers masked from the H10k and H2k high-density panels, respectively

### Comparison

The hybrid method was compared to the AlphaImpute and MaCH imputation methods using simulated and real data. The comparison was made in terms of computation time and imputation accuracy for different imputation strategies. Final results for the comparison of the simulated data are presented as the mean of five replicates.

The computation time was measured as the CPU time that the three methods used to run the simulated data. The CPU time is the total amount of time the CPU spent executing instructions. If the software were run in serial mode, the CPU time would be comparable to the real time the software takes to run. However, AlphaImpute and the hybrid method allow parallelization of some calculations. For instance, the phasing runs of the long-range phasing step of AlphaImpute and the hybrid method can be run simultaneously. Also the updating process of an individual’s haplotypes of the hybrid method is independent for each animal and can be run in parallel.

The imputation accuracy was computed as the average of animal-wise Pearson correlations between the true and imputed allele dosages [24]. Explicitly this involved calculating the correlation between the true and imputed allele dosages for each animal and averaging these correlations across animals in a category. For the simulated data that were generated using the simulated pedigree, the average was computed over the 1000 individuals in the last generation when the pedigree information was available, and over the unrelated individuals without pedigree information otherwise. For the real data and the simulated data using the real pig pedigree, averages were computed over the 509 animals in the testing set but according to the six categories of animals grouped by their pattern of relationship to their most recent densely genotyped ancestors. For instance, the averaged imputation accuracy of the ‘Both’ category was computed among the 46 animals within that category. A summary of the number of animals per category is in Table 2.

**Table 2:** Number of animals per category

Category	Count
Both	46
SireMGS	63
DamPGS	21
Sire	36
Dam	19
Other	324
Total	509

Number of animals per category based on the relationship to their most recent densely genotyped ancestors: both parents genotyped (Both); sire and maternal grandsire (SireMGS); dam and paternal grandsire (DamPGS); sire only (Sire); dam only (Dam); and other relatives (Other)

The imputation accuracy was also computed as the average of marker-wise Pearson correlations between the true and imputed allele dosages. Averages were computed among genotypes categorized into intervals of minor allele frequencies: [0.0, 0.025], [0.025, 0.05], [0.05, 0.075], [0.075, 0.10], [0.10, 0.15], [0.15, 0.20], [0.20, 0.25], [0.25, 0.30], [0.30, 0.35], [0.35, 0.40], [0.40, 0.45], and [0.45, 0.50].

For the simulated data, several imputation strategies using different combinations of the number of the template haplotypes and the number of iterations of the HMM parameters were set. The number of template haplotypes ranged from 100 to 300, with increments of 50 haplotypes. The number of iterations ranged from 20 to 50 with increments of 10, with 5 burn-in iterations.

Other parameters for both the heuristic and the HMM were set by default and are described in the following section.

#### Parameter settings

For the heuristic methods, a default set of 10 core lengths ranging from 500 to 9000 high-density markers was used for the various iterations of the long-range phasing and haplotype library imputation process. The set of cores was chosen to minimize both the computational intensity of the phasing processes and to maximize the number of times an allele was phased as a part of cores spanning different markers. The number of processors available was set to 20 in order to run simultaneously all the phasing runs of the long-range phasing step of the hybrid method and AlphaImpute. The number 20 comes from two times for each of the 10 core lengths, as described in the original publication of AlphaImpute [3]. The number of iterations of the heuristic rules was set by default to 5 for both the hybrid method and AlphaImpute.

For the HMM, the thresholds that control the training and imputation modes of the hybrid method and the number of processors available were set by default. A threshold of 50% was chosen for the training mode. Thus, if more than 50% of the individuals were phased by the previous heuristic step, their phased gametes were used to populate the template haplotypes in the HMM. An individual is considered to be phased if 99% or more of its markers have been phased. A threshold of 90% was chosen for the imputation mode. Thus, if more than 90% of the markers of an individual were imputed by the previous heuristic step, then the haploid version of the HMM was used for that individual, otherwise the diploid version was used. The number of processors available for the HMM was set to 8. This is arbitrary and depends on the number of processors that are available. The number of burn-in iterations was set to 5.

## Results

We compared the computation time and imputation accuracy, of the hybrid method, AlphaImpute and MaCH. AlphaImpute had the fastest computation time, followed by the hybrid method and then MaCH. The hybrid method was the most accurate and its accuracy increased with the number of template haplotypes and with the marker density of the low-density panel. Removing pedigree information reduced imputation accuracy for the hybrid method and AlphaImpute, but the hybrid method remained the most accurate. The imputation accuracy of all three methods decreased with the minor allele frequency and the hybrid method was the most accurate across minor allele frequencies.

### Computation time

AlphaImpute always required the same CPU time under the parameter settings considered, whereas the CPU time required by the two HMM increased with the number of template haplotypes and with the number of iterations. AlphaImpute was the fastest, and the hybrid method was faster than MaCH regardless of the number of template haplotypes or the number of iterations.

**Fig. 1:**
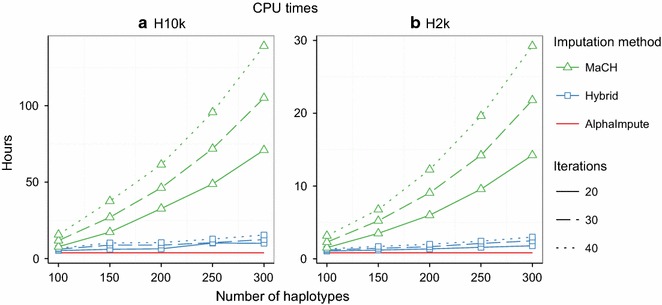
CPU times. Computation time in CPU hours for imputing to **a** the H10k and **b** the H2k high-density panels. Subfigures show CPU times of the hybrid method (*blue*), AlphaImpute (*red*) and MaCH (*green*) imputation methods for different numbers of template haplotypes and iterations (*different line styles*). AlphaImpute is independent of the number of template haplotypes and iterations, and is shown as a *horizontal line*

Figure 1 shows computation times, in CPU hours, of the three imputation methods for the number of template haplotypes and iterations. Real times are reported in Additional file 1: Figure S1. Panels (a) and (b) of Fig. 1 show the computation times for imputing to the H10k high-density panel (H10k) and H2k high-density panel (H2k), respectively. The computation time of AlphaImpute was independent of the number of template haplotypes and the number of iterations, whereas the computation time of the hybrid method and MaCH varied with the number of template haplotypes and iterations. The computation time of the hybrid method increased almost linearly with the number of template haplotypes whereas computation time of MaCH increased quadratically with the number of template haplotypes. This quadratic increase made MaCH the slowest method for large numbers of template haplotypes. The computation time of both the hybrid method and MaCH increased linearly with the number of iterations.

Computation times were measured using an Intel Xeon E5-2630 v3 (2.4 GHz) 16-cores processor based cluster running on a 64-bit Linux (Scientific Linux 7).

### Imputation accuracy

#### Simulated data

In this section, we compare the imputation accuracies of the three methods using different numbers of template haplotypes, varying low-density panels and for imputing with and without pedigree information. The main results can be summarized in five points:

1. When pedigree information was fully available, the hybrid method was comparable to AlphaImpute and better than MaCH.
2. When pedigree information was not available, the hybrid method was about twice as accurate as AlphaImpute and MaCH for the low-density panels.
3. When pedigree information was not available, the imputation accuracy of the hybrid method and MaCH increased with the number of template haplotypes.
4. The imputation accuracy of the hybrid method increased with the marker density of the low-density panels and was higher than the accuracy of AlphaImpute and MaCH across all the low-density panels.
5. The imputation accuracy of the hybrid method was the highest and remained stable for six categories of individuals depending on which of their immediate ancestor were genotyped at high-density.

**Fig. 2:**
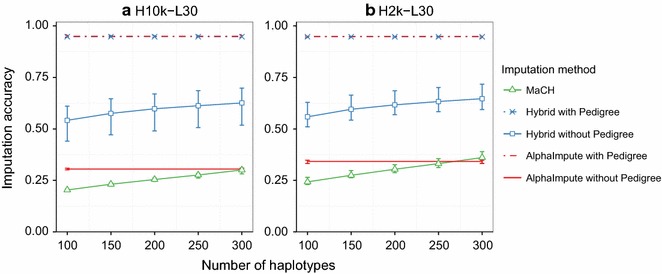
Imputation accuracy for the number of template haplotypes. Imputation accuracies for different numbers of template haplotypes corresponding to the imputation from the L30 low-density panel to **a** the H10k and **b** the H2k high-density panels. Within each plot the *different lines* show imputation accuracies of the hybrid method (*blue*), AlphaImpute (*red*) and MaCH (*green*) for different numbers of template haplotypes. Imputation was performed with (*dashed*) and without (*solid*) pedigree. The accuracy of AlphaImpute is independent of the number of template haplotypes and iterations, and is shown as a *horizontal line* across haplotypes

When pedigree information was available, the hybrid method was comparable to AlphaImpute and better than MaCH. Figure 2 shows the imputation accuracies of all three methods for different numbers of template haplotypes. Panels (a) and (b) of Fig. 2 show the imputation accuracies for imputing from the L30 low-density panel to the H10k high-density panel (H10k-L30) and from the L30 low-density panel to the H2k high-density panel (H2k-L30), respectively. The imputation accuracies of the hybrid method and AlphaImpute were 0.95 for imputing to both the H10k and H2k high-density panels, whereas the imputation accuracy of MaCH ranged from 0.20 to 0.35. Because MaCH is pedigree-free, we made a separate comparison of its imputation accuracy with those of the hybrid method and AlphaImpute without pedigree, as well.

Figure 2 also shows that without pedigree information, the imputation accuracy of AlphaImpute and MaCH was about 55% (H10k) and 47% (H2k) worse than that of the hybrid method, which increased with the number of template haplotypes. When pedigree information was not used, the imputation accuracy of the hybrid method ranged between 0.50 and 0.65, while imputation accuracy of MaCH ranged from 0.20 to 0.35, depending on the number of template haplotypes. The imputation accuracy of AlphaImpute is independent of the number of template haplotypes and its imputation accuracy was 0.31 and 0.34 for imputing from L30 to H2k and H10k high-density panels. For both high-density panels, the accuracies of AlphaImpute and MaCH were comparable if MaCH used more than 250 (H10k) and more than 200 (H2k) template haplotypes and were about half the accuracy of the hybrid method (>0.50).

Figure 2 shows that the imputation accuracies for imputing to the H10k high-density panel were always slightly less accurate than imputation to the L2k panel. For this reason, we consider only the H10k high-density panel hereinafter.

Next, we compared the imputation accuracies of the hybrid method and MaCH using an imputation of 200 template haplotypes over 20 iterations, which represents a good compromise between time and accuracy (see Additional file 2: Table S1).

**Fig. 3:**
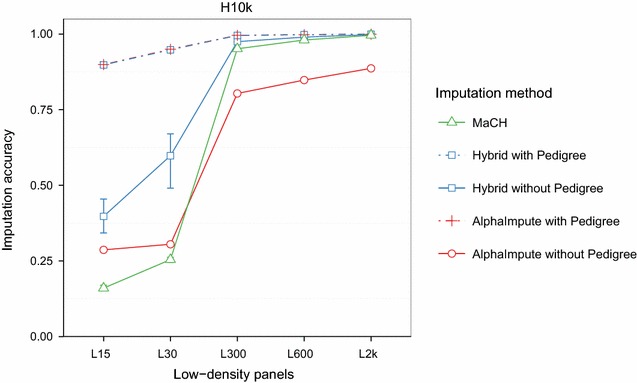
Imputation accuracies for different low-density panels. The imputation accuracy of the hybrid method (*blue*), AlphaImpute (*red*) and MaCH (*green*) were calculated for genotyping strategies corresponding to imputation from low-density panels to the H10k high-density panel. Imputation was performed with (*dashed*) and without (*solid*) pedigree

The imputation accuracy of the hybrid method increased with increased marker density of the low-density panels and exceeded the accuracy of AlphaImpute and MaCH when pedigree information was missing. Figure 3 shows the imputation accuracies from the low-density panels to H10k high-density panel. When the pedigree information was not used, the accuracies of all three methods showed a massive jump from the L30 to the L300 low-density panel and more gradual linear changes above that. Without pedigree information, the hybrid method was always the most accurate with imputation accuracies above 0.97 for the three highest low-density panels (L300, L600 and L2k), MaCH was nearly as accurate and AlphaImpute was 15 to 20% worse. For the lowest low-density panels (L15 and L30), the hybrid method was the most accurate method followed by AlphaImpute and then MaCH when pedigree information was missing. When pedigree information was available, the hybrid method and AlphaImpute were the most accurate with imputation accuracies above 0.99 for the three highest low-density panels and above 0.90 for the lowest low-density panels.

**Fig. 4:**
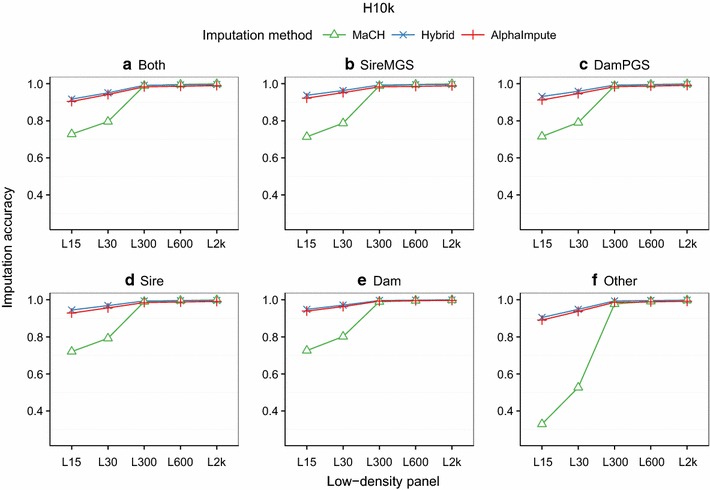
Imputation accuracy in simulated data for low-density panels across categories of genotype information available on immediate ancestors. Imputation accuracies of the hybrid method (*blue*), AlphaImpute (*red*) and MaCH (*green*) for low-density panels and for six categories of animals based on which immediate ancestor was genotyped at high-density: both parents genotyped (Both); sire and maternal grandsire (SireMGS); dam and paternal grandsire (DamPGS); sire only (Sire); dam only (Dam); and other relatives (Other). The categories depend on the relationship of the animals to their most recent densely genotyped ancestors

The imputation accuracy of all three methods increased with increased marker density of the low-density panels for different levels of genotype information available on immediate ancestors. Figure 4 shows imputation accuracy of all three methods plotted against the marker density of the low-density panel and the H10k high-density panel for simulated data and for six categories of individuals labeled according to which immediate ancestor was genotyped at high-density. Identical results for the H2k high-density panel are provided in Additional file 3: Figure S2. The imputation accuracies of all the three methods were similar and stable (>0.97) across varying levels of genotype information available on immediate ancestors for the three highest low-density panels. For the L15 low-density panel, the imputation accuracy of the hybrid and AlphaImpute decreased up to 9.5% and that of MaCH up to 66% with respect the L300 low-density panel. However, this reduction in imputation accuracy varied across different categories.

Reducing the amount of genotype information available on immediate ancestors affected the imputation accuracy. Panels (a)–(f) of Fig. 4 show imputation accuracies of all the three methods for the categories where both parents (Both), sire and maternal grandsire (SireMGS), dam and paternal grandsire (DamPGS), sire only (Sire), dam only (Dam), and other relatives (Other) were genotyped at high-density, respectively. The imputation accuracy of the hybrid and AlphaImpute was higher than 0.90 across all the categories for the two lowest low-density panels. The imputation accuracy of MaCH decreased to less than 0.73 (L15) when at least one of the parents was genotyped at high-density and dropped to 0.33 (L15) when none of the parents were genotyped at high-density (Other).

#### Real data

This section shows a comparison of the imputation accuracies of all three methods when real data was used (see Additional file 4: Table S2). For the comparison of the hybrid method and MaCH, we considered 200 template haplotypes and 20 estimation iterations as parameters of the HMM. The comparison provided two main results regarding the low-density panels and the genotype information available on immediate ancestors.

**Fig. 5:**
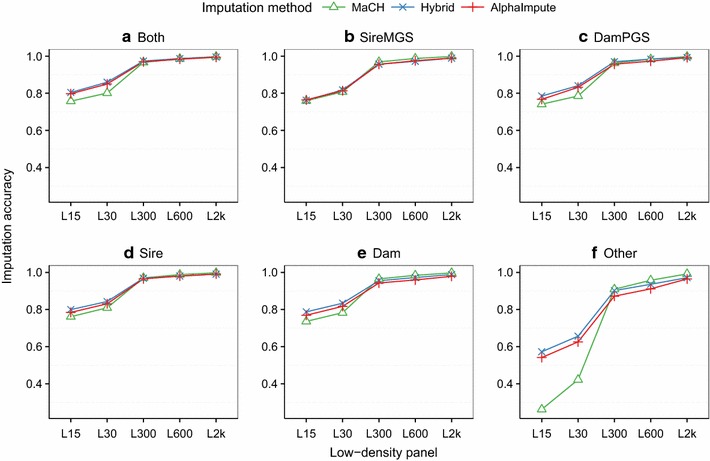
Imputation accuracy in real data for low-density panels across categories of genotype information that is available on immediate ancestors. Imputation accuracies of the hybrid method (*blue*), AlphaImpute (*red*) and MaCH (*green*) for low-density panels and for six categories of animals based on which immediate ancestor was genotyped at high-density: both parents genotyped (Both); sire and maternal grandsire (SireMGS); dam and paternal grandsire (DamPGS); sire only (Sire); dam only (Dam); and other relatives (Other)

The imputation accuracy of all three methods increased with increased marker density of the low-density panels for different categories of available genotype information on immediate ancestors. Figure 5 shows the imputation accuracy of all three methods plotted against the marker density of the low-density panel for real data and for animals that differ based on which close ancestor was densely genotyped. The imputation accuracies of the three methods were similar (>0.90) for the three highest low-density panels and for all the categories. For the lowest low-density panels, the hybrid method and AlphaImpute performed marginally better than MaCH (>0.80) when at least one parent was genotyped at high-density.

Reducing the amount of genotype information of both parents decreased the imputation accuracy. Panels (a)–(f) of Fig. 5 show imputation accuracies of all three methods for the categories where both parents (Both), sire and maternal grandsire (SireMGS), dam and paternal grandsire (DamPGS), sire only (Sire), dam only (Dam), and other relatives (Other) were genotyped at high-density, respectively. The imputation accuracies of the three methods were above 0.95 for the highest low-density panels and slightly decreased when none of the parents was genotyped at high-density (Other). For low-density panels with the lowest marker density, the imputation accuracies decreased from ~0.8 across all categories to ~0.6 for the hybrid method and AlphaImpute, and to less than 0.4 for MaCH when none of the parents were genotyped at high-density (Other).

### Minor allele frequency

In this section, we compare the imputation accuracies of the three imputation methods against the minor allele frequency in simulated data. Two hundred template haplotypes and 20 iterations were used as parameters of the HMM. The results showed:

1. the imputation accuracy of the three methods increased with increases in the minor allele frequency,
2. the imputation accuracy of the three methods increased as the genotype information of the low-density panels increased, and
3. the hybrid method always had the greatest accuracy when pedigree information was missing.

#### Imputation accuracy in relation to minor allele frequency

**Fig. 6:**
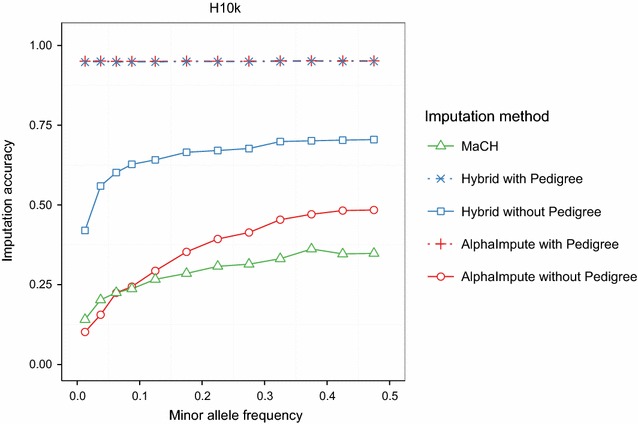
Imputation accuracies for different minor allele frequency values. Imputation accuracies for different minor allele frequency values of the marker being imputed for imputation from the L30 low-density panel to the H10k. The figure shows the imputation accuracy of the hybrid method (*blue*) and AlphaImpute (*red*) and MaCH (*green*). Imputation with the hybrid method and AlphaImpute was performed with (*dotted*) and without (*solid*) pedigree. MaCH is pedigree-free. The imputation accuracies were computed among genotypes categorized into groups of allele frequencies in the following intervals: [0.0, 0.025], [0.025, 0.05], [0.05, 0.075], [0.075, 0.10], [0.10, 0.15], [0.15, 0.20], [0.20, 0.25], [0.25, 0.30], [0.30, 0.35], [0.35, 0.40], [0.40, 0.45], and [0.45, 0.50]

The hybrid method was always the most accurate imputation method across the full spectrum of minor allele frequencies when pedigree information was missing. Figure 6 shows the imputation accuracy of MaCH and those of the hybrid method and AlphaImpute with and without pedigree information for different minor allele frequencies. Figure 6 shows the imputation accuracies for imputing from the L30 low-density panel to the H10k high-density panel (H10k-L30). Similar results for the H2k high-density panel and the other low-density panels are provided in Additional file 5: Figure S3 and Additional file 6: Figure S4.

The hybrid method and AlphaImpute were the most accurate and their imputation accuracy remained above 0.94 across all minor allele frequencies when the pedigree was available. Figure 6 also shows that when the pedigree information was removed, the imputation accuracy of the three methods decreased with decreases in the minor allele frequency. In this case, the hybrid method was the most accurate and its accuracy increased gradually as the minor allele frequency increased. Its accuracy was always above 0.60 except for minor allele frequencies that were lower than 0.05 for which the accuracy dropped below 0.50. In the absence of pedigree information, AlphaImpute was more accurate than MaCH except for rare alleles with minor allele frequencies higher than 0.1. However, even at the highest minor allele frequencies MaCH never exceeded the accuracy attained by the hybrid method at the lowest minor allele frequencies.

**Fig. 7:**
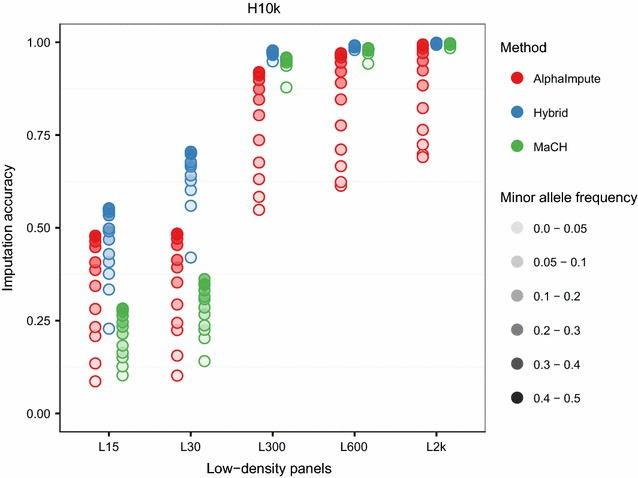
Imputation accuracies across minor allele frequencies for different low-density panels when pedigree information was removed. The imputation accuracies of the hybrid method (*blue*), AlphaImpute (*red*) and MaCH (*green*) were computed for imputations from a low-density panel to the H10K high-density panel. Different minor allele frequencies are represented with different opacities

Figure 7 shows the imputation accuracy of the hybrid method and AlphaImpute when pedigree information was missing for imputation from a low-density panel to the H10k panel as a function of the marker density of the low-density panels with minor allele frequency as a parameter. The imputation accuracy of the three methods increased with increased marker density of the low-density panels and, for any given method and marker density, accuracy increased with allele frequency. The hybrid method was always the most accurate. From moderate to high genotype densities of the low-density panels (L300, L600 and L2k), the accuracy of the hybrid method was above 0.95 for all allele frequencies in the absence of pedigree information. MaCH came second and had imputation accuracies above 0.85. AlphaImpute was the least accurate imputation method and was the most sensitive to minor allele frequency with accuracies below 0.75 for low values of minor allele frequencies when pedigree information was missing.

For the lowest marker density of the low-density panels (L15 and L30), the imputation accuracy of all three methods was substantially lower and more sensitive to allele frequency. The hybrid method was the most accurate for all values of minor allele frequency. In the absence of pedigree information, AlphaImpute was more accurate than MaCH for higher values of minor allele frequency, and slightly worse for very low values.

## Discussion

The results show that among those tested, the hybrid imputation method is the most accurate method at different genotype densities with or without pedigree information. It is also faster than MaCH and only slightly slower than AlphaImpute. These results raise four points for discussion:

1. What are the advantages of using the hybrid method versus using a faster and nearly as accurate imputation method like AlphaImpute?
2. How does the computation time of the hybrid method benefit from the combination of heuristic and probabilistic approaches?
3. What is the best way to measure imputation accuracy of genotypes with rare alleles?
4. How does the hybrid method benefit from the combination of heuristic and probabilistic approaches to impute genotypes with rare alleles?

The advantage of using the hybrid method although it is slower than AlphaImpute is its higher accuracy when pedigree information is missing. In addition, AlphaImpute fails to impute genotypes that do not meet the imputation heuristic rules even when pedigree information is fully available. This causes some genotypes and segments of haplotypes to remain unimputed after the imputation. The HMM implemented in the hybrid method overcomes this limitation and calls the genotype of all markers for all individuals in the population.

Imputation methods usually comprise a phasing step that resolves the haplotypes of individuals genotyped at high-density, and an imputation step that identifies which combinations of these haplotypes are carried by the individuals genotyped at low-density. The hybrid method creates a reference set of extremely accurate haplotypes by applying a long-range phasing and haplotype library heuristic imputation algorithm, AlphaPhase [4]. The advantage of AlphaPhase is that the phasing algorithm can use the genotype information of surrogate parents (i.e., individuals that share a haplotype with the proband and that do not have any opposing homozygous genotypes with the proband). This makes it unnecessary to genotype multiple generations of ancestors at high-density in order to phase genotypes. The hybrid method then uses these well-phased haplotypes to impute the missing genotypes in a combined heuristic and probabilistic approach.

In the imputation step, the hybrid method accurately imputes genotypes by identifying the exact pair of haplotypes for single markers or groups of consecutive markers with basic heuristic pedigree-based phasing rules [3]. However, the heuristic rules are not sufficient to model the recombination of those haplotypes. Failures are particularly pronounced for individuals that are genotyped at very low-density because it is very likely that recombination occurs at markers for which genotype information cannot be inferred, leading to haplotype switch errors. To avoid haplotype switch errors, the hybrid method uses the HMM of MaCH [13] to estimate the recombination rates between markers and the most likely position for recombination in a given haplotype.

The accurate pre-phasing helps to reduce the computation time of the hybrid method without loss in imputation accuracy by decreasing the number of iterations and the number of template haplotypes. This makes the hybrid method faster than similar HMM methods such as MaCH. Unlike MaCH, the hybrid method takes advantage of the pedigree information to pre-phase and unambiguously impute genotypes. Pre-phasing genotype data provides very accurate haplotypes to be used by the HMM. If the number of well-phased haplotypes is high, the faster haploid version of the HMM can be used in most cases instead of the slower diploid version. In fact, our results show how the computation time of the hybrid method scales almost linearly with the number of template haplotypes, whereas the computation time of MaCH scales quadratically. Moreover, seeding a probabilistic method with well-phased haplotypes means that the model parameters are estimated with fewer iterations. Also, the hybrid method is more robust than MaCH in terms of the number of template haplotypes especially when pedigree information is available (see Additional file 7: Figure S5, Additional file 8: Figure S6). By speeding up the HMM via integration into the hybrid method, it can be applied to larger datasets and its own inherent accuracy can be higher since a user can use more template haplotypes for a fixed amount of computation time. More template haplotypes make the HMM more accurate.

Correct quantification of imputation accuracy of genotypes with rare alleles is especially important for sequence data for which there are many alleles with low frequencies, and the marker-wise correlation between the true genotypes and allele dosages is the best way to measure this imputation accuracy. Imputation accuracy computed in this way measures how much is gained by a sophisticated imputation method in contrast to naïve procedures of imputing two times the allele frequency or the most frequent genotype. The correlation between true genotypes and genotype dosages is a better estimator of imputation accuracy than imputation error rates, particularly for the imputation of genotypes with very rare alleles (<0.1). Imputation error rate, computed as the percentage of genotypes that are imputed incorrectly is strongly affected by the minor allele frequency [24, 25]. When the minor allele frequency is very low, missing genotypes are almost certain to be homozygous for the common allele and naïve imputation methods yield an imputation error rate of almost 0%. The prior uncertainty increases with minor allele frequency, and thus imputation error rate increases as the minor allele frequency increases [25]. In contrast, the correlation coefficient between true genotypes and the allele dosages is an unbiased estimator of the imputation accuracy [24]. The Pearson correlation coefficient (used to calculate imputation accuracy in this paper) assumes that the two variables are normally distributed and requires that the mean and variance be standardized for both true genotypes and genotype dosages in order to calculate the correlation across individuals for a single locus. Thus, true genotypes and dosages were normalized by the mean and standard deviation of the true values. As a consequence, loci with a low minor allele frequency, for which genotypes are difficult to impute, are given more importance.

Marker-wise imputation accuracies of the hybrid method show that the combination of heuristic methods with a HMM helps to impute genotypes with rare alleles. Our results demonstrate that probabilistic methods based on linkage disequilibrium such as the HMM in MaCH are less accurate than heuristic methods based on linkage such as AlphaImpute, which agrees with previous studies [13, 24, 25]. However, the hybrid method was more accurate than both AlphaImpute and MaCH across minor allele frequencies and across genotype densities especially when pedigree information was missing (see Additional file 5: Figure S3). A possible explanation for this is that rare alleles become more frequent in the template haplotypes used by the HMM because heuristic methods are able to impute some of these rare alleles without ambiguity.

## Conclusions

The hybrid heuristic and probabilistic imputation method proposed in this paper imputes accurate genotypes quickly for large populations and large numbers of markers even when limited pedigree information is available. It is usually more accurate and never significantly less accurate than a purely heuristic method and is faster than a standard probabilistic method.

## Additional files

**Additional file 1: Figure S1.** Real computation time in hours for imputing to **a** the H10k and **b** the H2k high-density panels. Subfigures show real times of the hybrid method (*blue*), AlphaImpute (*red*) and MaCH (*green*) imputation methods for different numbers of template haplotypes and iterations (*different line styles*). AlphaImpute is independent of the number of template haplotypes and iterations, and is shown as a *horizontal line*.

**Additional file 2: Table S1.** Summary of imputation accuracies. Imputation accuracy for different imputation from L15, L30, L300, L600 and L2k low-density panels to H2k and H10k high-density panels. Imputation accuracies of the hybrid method and AlphaImpute were computed for imputation performed with and without pedigree. MaCH is always pedigree-free. Imputation accuracies of the hybrid method and MaCH correspond to a parameter setting that is equal to 200 template haplotypes and 20 iterations.

**Additional file 3: Figure S2.** Imputation accuracy in simulated data for different low-density panels across categories of genotype information available on immediate ancestors. Imputation accuracies of the hybrid method (*blue*), AlphaImpute (*red*) and MaCH (*green*) for different low-density panels and for six categories of animals according to which of their immediate ancestor are genotyped at high-density: both parents genotyped (Both); sire and maternal grandsire (SireMGS); dam and paternal grandsire (DamPGS); sire only (Sire); dam only (Dam); and other relatives (Other). The categories depend on the relationship of the animals to their most recent densely genotyped ancestors.

**Additional file 4: Table S2.** Summary of imputation accuracies for the real data. Imputation accuracy calculated as the correlation between the true genotypes and the genotype dosages across different categories of animals in the testing set. Animals in the testing set were grouped into six different categories according to which of their immediate ancestors are genotyped at high-density: both parents genotyped (Both); sire and maternal grandsire (SireMGS); dam and paternal grandsire (DamPGS); sire only (Sire); dam only (Dam); and other relatives (Other). Imputation accuracy of the hybrid method and MaCH correspond to a parameter setting that is equal to 200 template haplotypes and 20 iterations.

**Additional file 5: Figure S3.** Imputation accuracies according to minor allele frequency when pedigree information was removed. Imputation accuracies according to minor allele frequency of the marker being imputed when pedigree information was removed for 500 of the 1000 individuals in the last generation. The imputation accuracy of the hybrid method (*blue*), AlphaImpute (*red*) and MaCH (*green*) are plotted in different subfigures corresponding to the imputation strategies from the L15, L30, L300, L600 and L2k low-density panels to the H10k (**a**–**e**) and H2k (**f**–**i**) high-density panels. The imputation accuracies were computed among genotypes categorized into groups of allele frequencies in the following intervals: [0.0, 0.025], [0.025, 0.05], [0.05, 0.075], [0.075, 0.10], [0.10, 0.15], [0.15, 0.20], [0.20, 0.25], [0.25, 0.30], [0.30, 0.35], [0.35, 0.40], [0.40, 0.45], and [0.45, 0.50].

**Additional file 6: Figure S4.** Imputation accuracies according to minor allele frequency of the marker being imputed when the pedigree information was fully available. The imputation accuracy of the hybrid method (*blue*), AlphaImpute (*red*) and MaCH (*green*) are plotted in different subfigures corresponding to the imputation strategies from the L15, L30, L300, L600 and L2k low-density panels to the H10k (**a**–**e**) and H2k (**f**–**i**) high-density panels. The imputation accuracies were computed among genotypes categorized into groups of allele frequencies in the following intervals: [0.0, 0.025], [0.025, 0.05], [0.05, 0.075], [0.075, 0.10], [0.10, 0.15], [0.15, 0.20], [0.20, 0.25], [0.25, 0.30], [0.30, 0.35], [0.35, 0.40], [0.40, 0.45], and [0.45, 0.50].

**Additional file 7: Figure S5.** Imputation accuracies of all three methods for different numbers of template haplotypes when pedigree information was removed. The different subfigures correspond to the imputation strategies from the L15, L30, L300, L600 and L2k low-density panels to the H10k (**a**–**e**) and H2k (**f**–**i**) high-density panels. Each subfigure plots imputation accuracies of the hybrid method (*blue*), AlphaImpute (*red*) and MaCH (*green*) for different numbers of template haplotypes. AlphaImpute is independent of number of template haplotypes and iterations and is shown as a *horizontal line* across haplotypes.

**Additional file 8: Figure S6.** Imputation accuracies of all three methods for different numbers of template haplotypes when pedigree information was fully available. The different subfigures correspond to the imputation strategies from the L15, L30, L300, L600 and L2k low-density panels to the H10k (**a**–**e**) and H2k (**f**–**i**) high-density panels. Each subfigure plots imputation accuracies of the hybrid method (*blue*), AlphaImpute (*red*) and MaCH (*green*) for different number of template haplotypes. AlphaImpute is independent of the number of template haplotypes and iterations and is shown as a *horizontal line* across haplotypes.
