# Supplementary figures and images for: A hybrid method for the imputation of genomic data in livestock populations
Source: Genet Sel Evol. 2017 Mar 3;49:30. doi: 10.1186/s12711-017-0300-y (PMC5439152; doi:10.1186/s12711-017-0300-y)

# Real times

a) H10k

b) H2k

Imputation method

- MaCH
- Hybrid
- AlphaImpute

Iterations

- 20
- 30
- 40

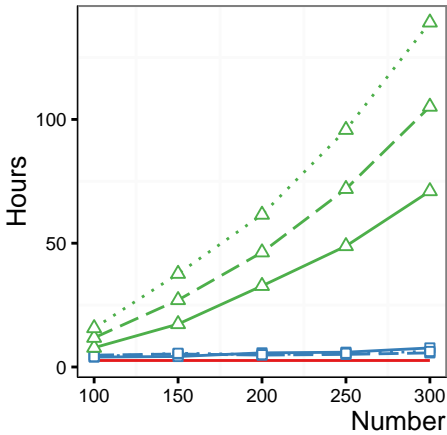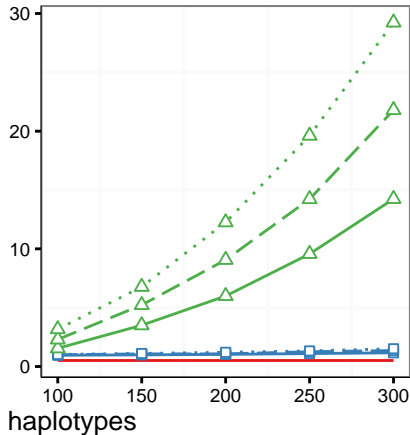

Supplement: Supplementary file 1 — Additional file 1: Figure S1. Real computation time in hours for imputing to a the H10k and b the H2k high-density panels. Subfigures show real times of the hybrid method (blue), AlphaImpute (red) and MaCH (green) imputation methods for different numbers of template haplotypes and iterations (different line styles). AlphaImpute is independent of the number of template haplotypes and iterations, and is shown as a horizontal line. [file 12711_2017_300_MOESM1_ESM.pdf]

## Without Pedigree

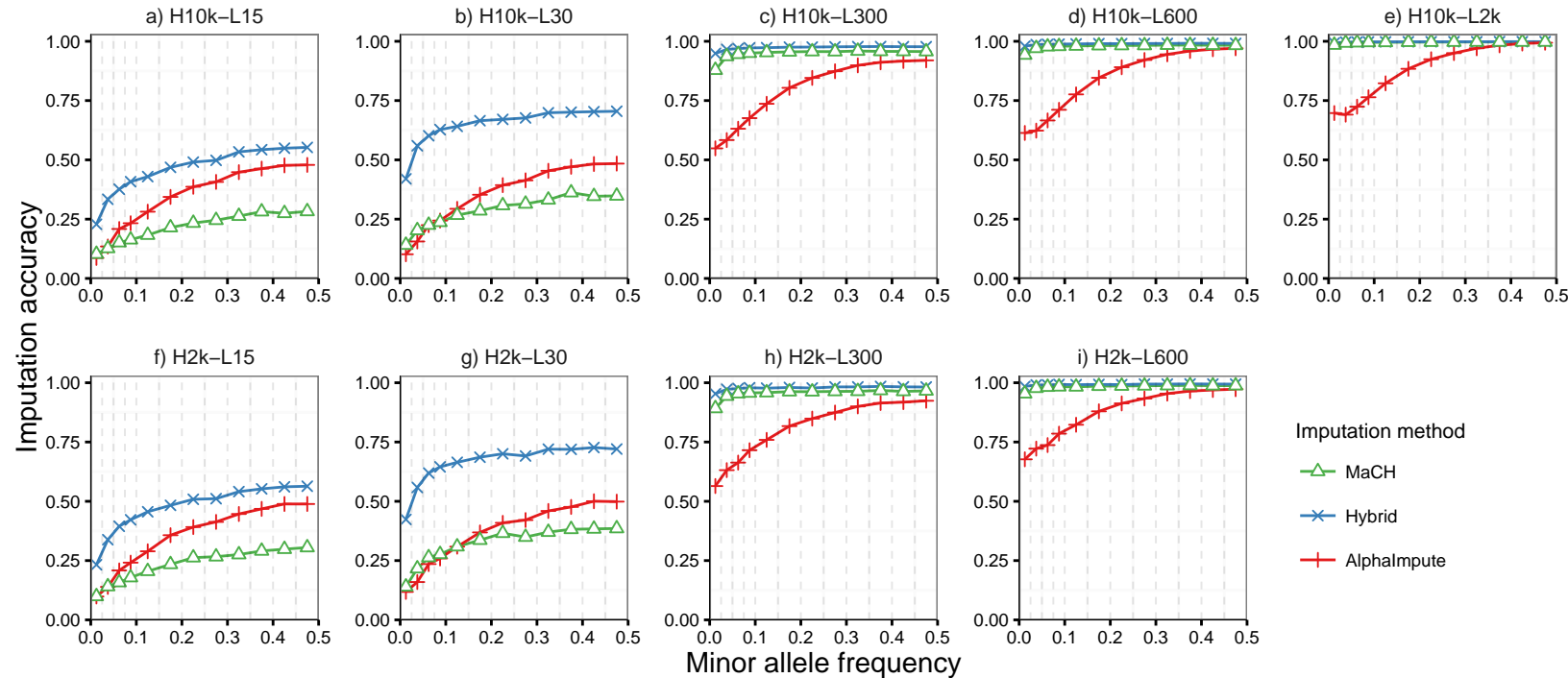

Supplement: Supplementary file 5 — Additional file 5: Figure S3. Imputation accuracies according to minor allele frequency when pedigree information was removed. Imputation accuracies according to minor allele frequency of the marker being imputed when pedigree information was removed for 500 of the 1000 individuals in the last generation. The imputation accuracy of the hybrid method (blue), AlphaImpute (red) and MaCH (green) are plotted in different subfigures corresponding to the imputation strategies from the L15, L30, L300, L600 and L2k low-density panels to the H10k (a–e) and H2k (f–i) high-density panels. The imputation accuracies were computed among genotypes categorized into groups of allele frequencies in the following intervals: [0.0, 0.025], [0.025, 0.05], [0.05, 0.075], [0.075, 0.10], [0.10, 0.15], [0.15, 0.20], [0.20, 0.25], [0.25, 0.30], [0.30, 0.35], [0.35, 0.40], [0.40, 0.45], and [0.45, 0.50]. [file 12711_2017_300_MOESM5_ESM.pdf]

## With Pedigree

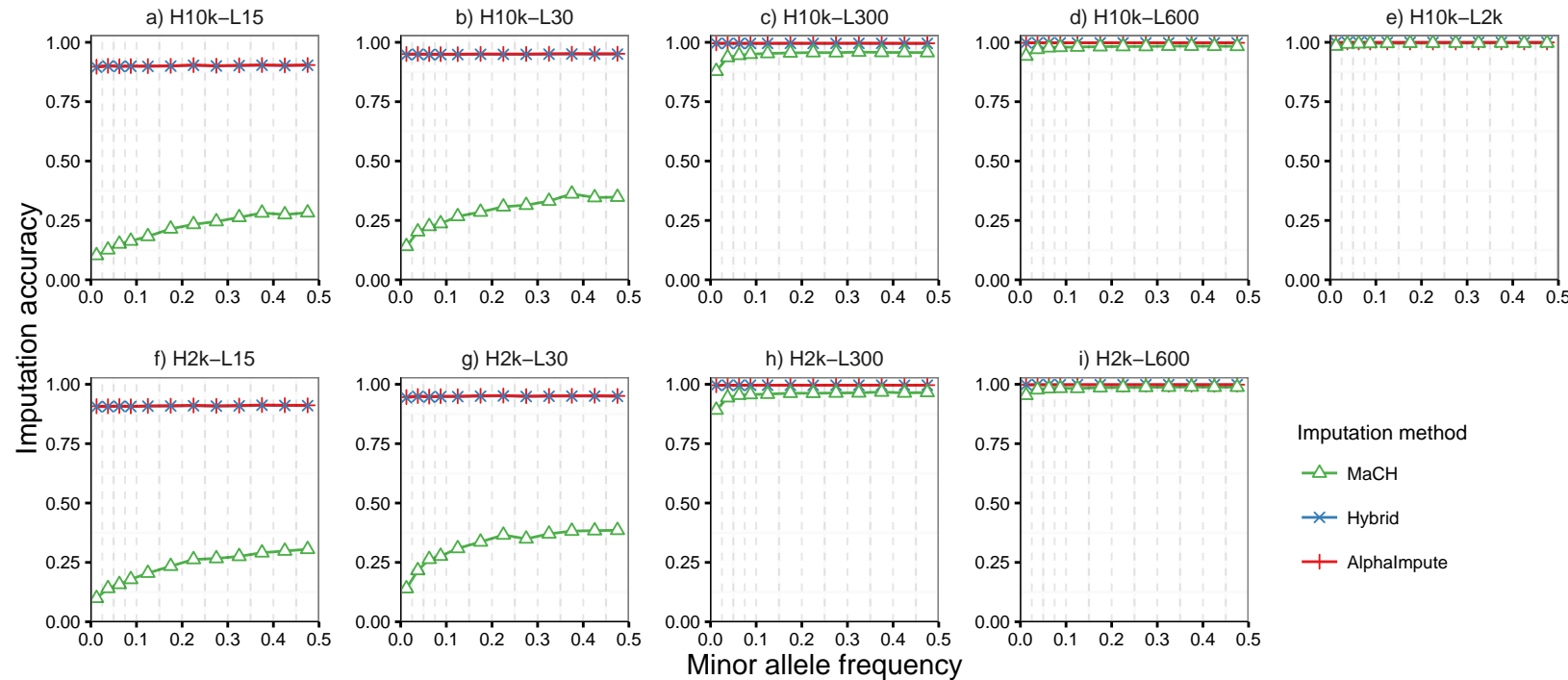

Supplement: Supplementary file 6 — Additional file 6: Figure S4. Imputation accuracies according to minor allele frequency of the marker being imputed when the pedigree information was fully available. The imputation accuracy of the hybrid method (blue), AlphaImpute (red) and MaCH (green) are plotted in different subfigures corresponding to the imputation strategies from the L15, L30, L300, L600 and L2k low-density panels to the H10k (a–e) and H2k (f–i) high-density panels. The imputation accuracies were computed among genotypes categorized into groups of allele frequencies in the following intervals: [0.0, 0.025], [0.025, 0.05], [0.05, 0.075], [0.075, 0.10], [0.10, 0.15], [0.15, 0.20], [0.20, 0.25], [0.25, 0.30], [0.30, 0.35], [0.35, 0.40], [0.40, 0.45], and [0.45, 0.50]. [file 12711_2017_300_MOESM6_ESM.pdf]

## Without Pedigree

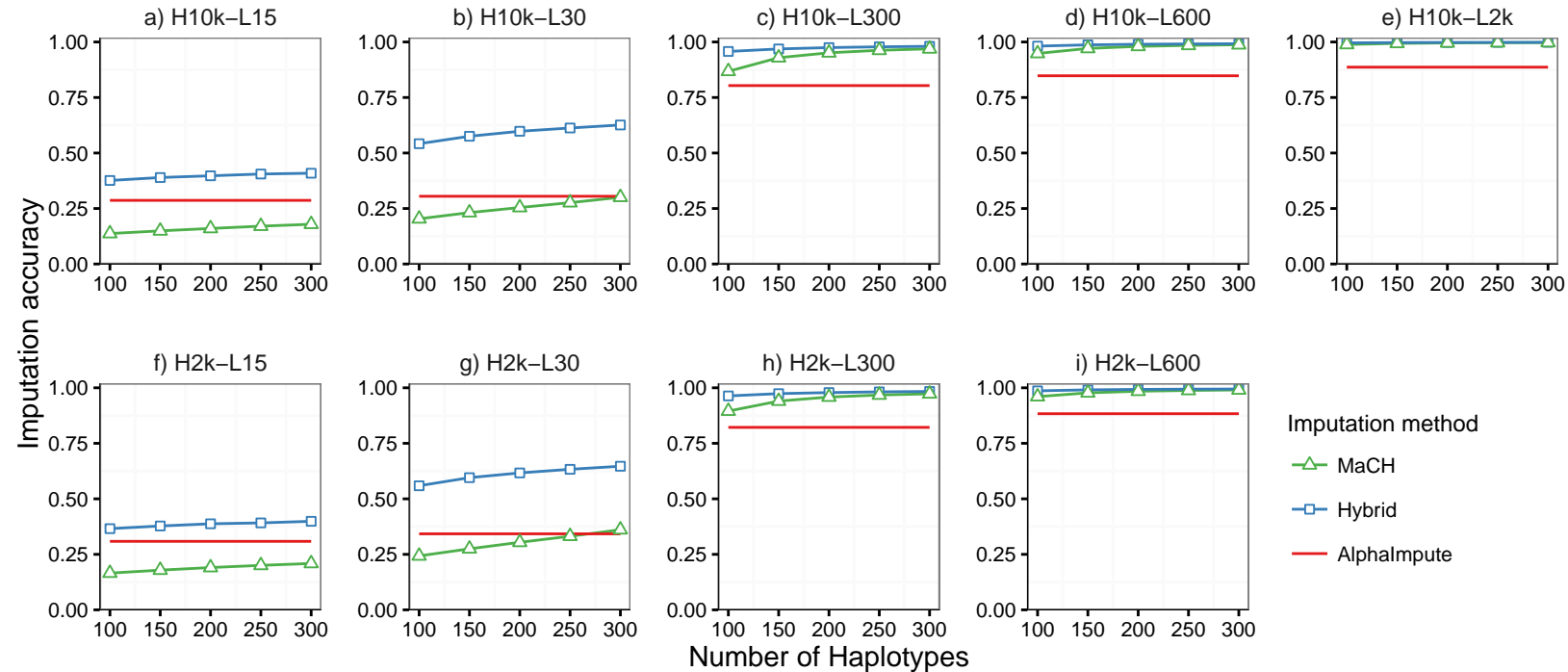

Supplement: Supplementary file 7 — Additional file 7: Figure S5. Imputation accuracies of all three methods for different numbers of template haplotypes when pedigree information was removed. The different subfigures correspond to the imputation strategies from the L15, L30, L300, L600 and L2k low-density panels to the H10k (a–e) and H2k (f–i) high-density panels. Each subfigure plots imputation accuracies of the hybrid method (blue), AlphaImpute (red) and MaCH (green) for different numbers of template haplotypes. AlphaImpute is independent of number of template haplotypes and iterations and is shown as a horizontal line across haplotypes. [file 12711_2017_300_MOESM7_ESM.pdf]

## With Pedigree

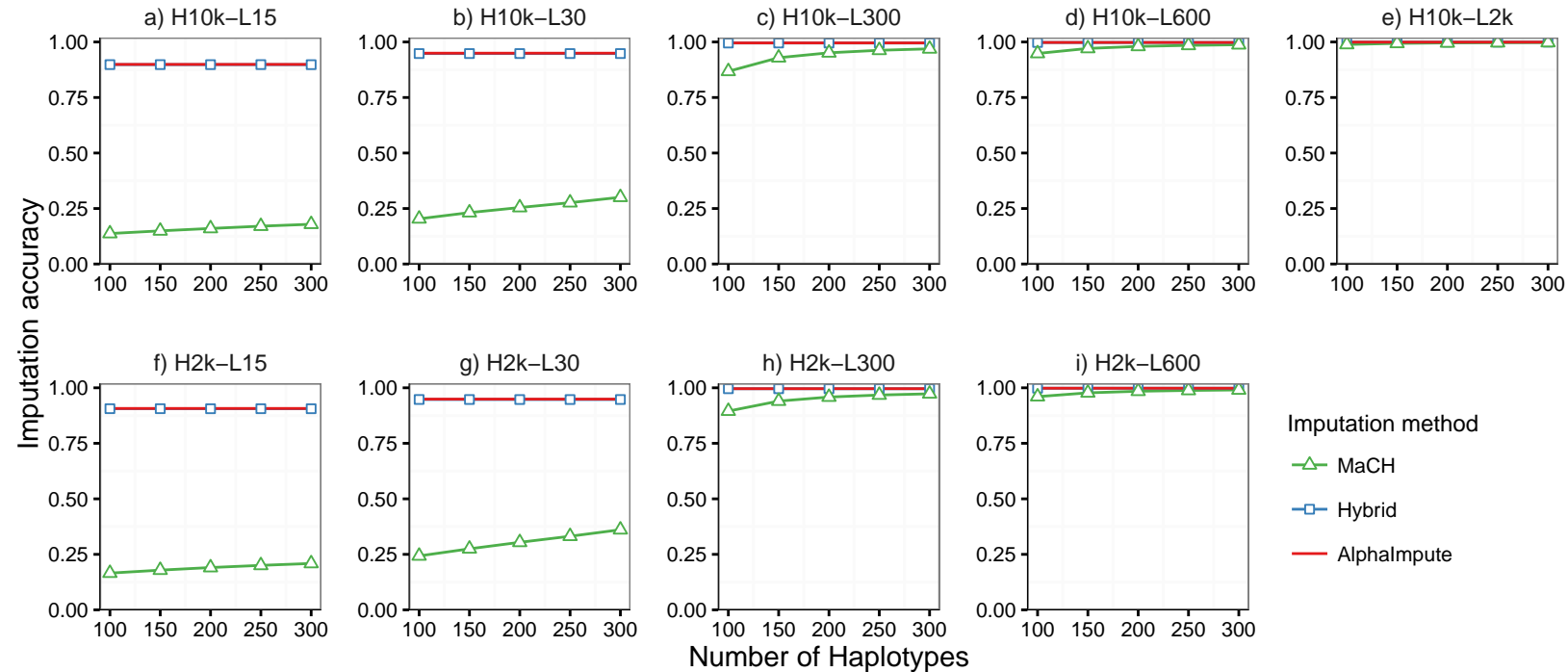

Supplement: Supplementary file 8 — Additional file 8: Figure S6. Imputation accuracies of all three methods for different numbers of template haplotypes when pedigree information was fully available. The different subfigures correspond to the imputation strategies from the L15, L30, L300, L600 and L2k low-density panels to the H10k (a–e) and H2k (f–i) high-density panels. Each subfigure plots imputation accuracies of the hybrid method (blue), AlphaImpute (red) and MaCH (green) for different number of template haplotypes. AlphaImpute is independent of the number of template haplotypes and iterations and is shown as a horizontal line across haplotypes. [file 12711_2017_300_MOESM8_ESM.pdf]
