# Supplementary material for: A hybrid method for the imputation of genomic data in livestock populations
Source: Genet Sel Evol. 2017 Mar 3;49:30. doi: 10.1186/s12711-017-0300-y (PMC5439152; doi:10.1186/s12711-017-0300-y)
Supplement: Supplementary file 2 — Additional file 2: Table S1. Summary of imputation accuracies. Imputation accuracy for different imputation from L15, L30, L300, L600 and L2k low-density panels to H2k and H10k high-density panels. Imputation accuracies of the hybrid method and AlphaImpute were computed for imputation performed with and without pedigree. MaCH is always pedigree-free. Imputation accuracies of the hybrid method and MaCH correspond to a parameter setting that is equal to 200 template haplotypes and 20 iterations. [file 12711_2017_300_MOESM2_ESM.docx]

## Additional Table S1 - Summary of imputation accuracies

|  |  |  | **Imputation strategy** | | | | | | | | |  |
| --- | --- | --- | --- | --- | --- | --- | --- | --- | --- | --- | --- | --- |
|  | Imputation to | **H10k** | | | | | |  | **H2k** | | | |
|  | Imputation from | **L2k** | | **L600** | **L300** | **L30** | **L15** |  | **L600** | **L300** | **L30** | **L15** |
| **Method** | **Pedigree Information** |  | |  |  |  |  |  |  |  |  |  |
| Hybrid | with Pedigree | 0.999 | | 0.998 | 0.995 | 0.948 | 0.898 |  | 0.998 | 0.996 | 0.947 | 0.906 |
|  | without Pedigree | 0.998 | | 0.990 | 0.975 | 0.598 | 0.397 |  | 0.993 | 0.979 | 0.617 | 0.388 |
| AlphaImpute | with Pedigree | 0.999 | | 0.998 | 0.996 | 0.949 | 0.899 |  | 0.998 | 0.996 | 0.951 | 0.907 |
|  | without Pedigree | 0.886 | | 0.847 | 0.803 | 0.305 | 0.287 |  | 0.883 | 0.821 | 0.341 | 0.309 |
| MaCH | - | 0.996 | | 0.980 | 0.951 | 0.255 | 0.160 |  | 0.984 | 0.958 | 0.306 | 0.191 |

Imputation accuracy for different imputation from L15, L30, L300, L600 and L2k low-density panels to H2k and H10k high-density panels. Imputation accuracies of the hybrid method and AlphaImpute were computed for imputation performed with and without pedigree. MaCH is always pedigree free. Imputation accuracies of the hybrid method and MaCH correspond to a parameter setting equal to 200 template haplotypes and 20 iterations.
