# Supplementary material for: A hybrid method for the imputation of genomic data in livestock populations
Source: Genet Sel Evol. 2017 Mar 3;49:30. doi: 10.1186/s12711-017-0300-y (PMC5439152; doi:10.1186/s12711-017-0300-y)
Supplement: Supplementary file 3 — Additional file 3: Figure S2. Imputation accuracy in simulated data for different low-density panels across categories of genotype information available on immediate ancestors. Imputation accuracies of the hybrid method (blue), AlphaImpute (red) and MaCH (green) for different low-density panels and for six categories of animals according to which of their immediate ancestor are genotyped at high-density: both parents genotyped (Both); sire and maternal grandsire (SireMGS); dam and paternal grandsire (DamPGS); sire only (Sire); dam only (Dam); and other relatives (Other). The categories depend on the relationship of the animals to their most recent densely genotyped ancestors. [file 12711_2017_300_MOESM3_ESM.pdf]

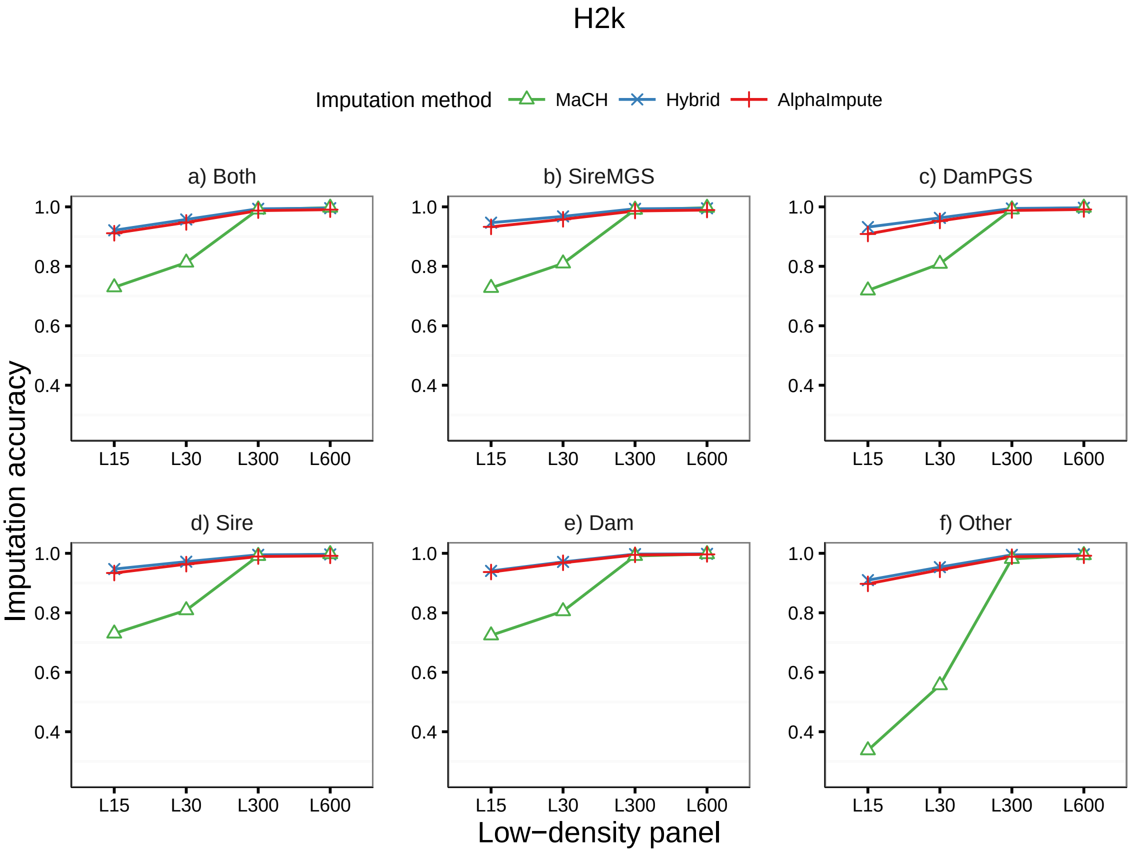
H2k
Imputation method
MaCH
Hybrid
AlphaImpute
a) Both
b) SireMGS
c) DamPGS
d) Sire
e) Dam
f) Other
1.0
0.8
0.6
0.4
L15
L30
L300
L600
Imputation accuracy
Low−density panel
