# Supplementary material for: A hybrid method for the imputation of genomic data in livestock populations
Source: Genet Sel Evol. 2017 Mar 3;49:30. doi: 10.1186/s12711-017-0300-y (PMC5439152; doi:10.1186/s12711-017-0300-y)
Supplement: Supplementary file 4 — Additional file 4: Table S2. Summary of imputation accuracies for the real data. Imputation accuracy calculated as the correlation between the true genotypes and the genotype dosages across different categories of animals in the testing set. Animals in the testing set were grouped into six different categories according to which of their immediate ancestors are genotyped at high-density: both parents genotyped (Both); sire and maternal grandsire (SireMGS); dam and paternal grandsire (DamPGS); sire only (Sire); dam only (Dam); and other relatives (Other). Imputation accuracy of the hybrid method and MaCH correspond to a parameter setting that is equal to 200 template haplotypes and 20 iterations. [file 12711_2017_300_MOESM4_ESM.docx]

## Additional Table S2 - Summary of imputation accuracies for the real data

|  |  |  | **Imputation strategy** | | | | |
| --- | --- | --- | --- | --- | --- | --- | --- |
| **Method** | **Pedigree information** | **Count** | **L2k** | **L600** | **L300** | **L30** | **L15** |
| *Hybrid* | Both | 46 | 1.00 | 0.99 | 0.97 | 0.86 | 0.80 |
|  | SireMGS | 63 | 0.99 | 0.97 | 0.96 | 0.82 | 0.76 |
|  | DamPGS | 21 | 0.99 | 0.98 | 0.97 | 0.84 | 0.79 |
|  | Sire | 36 | 0.99 | 0.98 | 0.97 | 0.84 | 0.80 |
|  | Dam | 19 | 0.99 | 0.97 | 0.96 | 0.83 | 0.79 |
|  | Other | 324 | 0.97 | 0.94 | 0.90 | 0.66 | 0.57 |
| *AlphaImpute* | Both | 46 | 0.99 | 0.98 | 0.97 | 0.85 | 0.80 |
|  | SireMGS | 63 | 0.99 | 0.97 | 0.96 | 0.81 | 0.76 |
|  | DamPGS | 21 | 0.99 | 0.97 | 0.96 | 0.83 | 0.77 |
|  | Sire | 36 | 0.99 | 0.98 | 0.97 | 0.83 | 0.78 |
|  | Dam | 19 | 0.98 | 0.96 | 0.94 | 0.82 | 0.77 |
|  | Other | 324 | 0.96 | 0.91 | 0.87 | 0.63 | 0.54 |
| *MaCH* | Both | 46 | 1.00 | 0.99 | 0.97 | 0.80 | 0.76 |
|  | SireMGS | 63 | 1.00 | 0.99 | 0.97 | 0.81 | 0.76 |
|  | DamPGS | 21 | 1.00 | 0.98 | 0.96 | 0.79 | 0.74 |
|  | Sire | 36 | 1.00 | 0.99 | 0.97 | 0.81 | 0.76 |
|  | Dam | 19 | 1.00 | 0.98 | 0.97 | 0.78 | 0.74 |
|  | Other | 324 | 0.99 | 0.96 | 0.91 | 0.42 | 0.26 |

Imputation accuracy calculated as the correlation between the true genotypes and the genotype dosages across different categories of animals in the testing set. Animals in the testing set were grouped into six different categories according to which of their immediate ancestor are genotyped at high-density: both parents genotyped (Both); sire and maternal grandsire (SireMGS); dam and paternal grandsire (DamPGS); sire only (Sire); dam only (Dam); and other relatives (Other). Imputation accuracy of the hybrid method and MaCH correspond to a parameter setting equal to 200 template haplotypes and 20 iterations.
